# Supplementary material for: Below the canopy: global trends in forest vertebrate populations and their drivers
Source: Proc Biol Sci. 2020 Jun 3;287(1928):20200533. doi: 10.1098/rspb.2020.0533 (PMC7341944; doi:10.1098/rspb.2020.0533)
Supplement: Further information on data sources and results [file rspb20200533supp1.pdf]

Below the canopy: global trends in forest vertebrate populations and their drivers.

Elizabeth J. Green, Louise McRae, Robin Freeman, Mike B.J. Harfoot, Samantha L.L. Hill, William Baldwin-Cantello, William D. Simonson.

Proceedings of the Royal Society B. DOI: 10.1098/rspb. 2020-0533

Supplementary Material

S1. Number of species and populations in the forest specialist index by realm and taxonomic class

	Afrotropical		Indopacific		Nearctic		Neotropical		Palearctic	
	Species	Populations	Species	Populations	Species	Populations	Species	Populations	Species	Populations
Birds	1	1	27	44	45	47	60	75	8	42
Mammals	27	49	19	32	6	32	31	54	6	14
Reptiles and amphibians	5	5	7	11	4	11	27	37	0	0

S2. Specifications of some global tree cover datasets with continuous vegetation fields

**Dataset name**	**Definition of tree cover**	**Spatial resolution**	**Temporal coverage/ cadence**	**Reference**
Landsat Global Forest Change	Canopy closure for all vegetation taller than 5 meters in height.	30 m	Fractional tree cover available for 2000 and 2010 only; tree cover loss available for 2001-2017	Hansen et al (2013)
Landsat VCF (GLCF)	The percentage of horizontal ground in each 30-m pixel covered by woody vegetation greater than 5 meters in height	30 m	2000, 2005, 2010	(Sexton et al., 2013)
MEASURES VCF5KYR	The proportion of the ground covered by the vertical projection of tree crowns. Trees are defined as all vegetation taller than 5 meters in height.	0.05 degree (5600 m)	1982-2016, annual	Song et al (2018)
MODIS VCF	Percent of the pixel covered by tree canopy equal to or greater than 5 m in height.	250 m	2000-2015, annual	(DiMiceli et al., 2015)

S3. Calculation of 5-km radius

Range size estimates were not known for every species, but body mass estimates were available (see *Additional drivers of forest population change* in main text). We calculated the correlation between body mass and range size (both log-10 transformed) for the species with both estimates available, and found a strong positive relationship (Pearson correlation coefficient = 0.87, $p < 0.001$). We therefore used body mass to predict range size for all populations using the ‘predict.lm’ function in the ‘stats’ package (R Core Team, 2018) and calculated the mean range size across all populations as 58.5 km^2, equivalent to a circle with a radius of 4.32 km. We rounded this up and fitted buffers with a 5-km radius around the central coordinates of each forest population.

S4. Number of forest populations (generalists and specialists) and forest specialist populations per realm included in analyses using long-term and short-term tree cover datasets.

Analysis	Realm	All forest populations	Forest specialist populations
Long-term (Song)	Afrotropical	365	36
	Australasia	64	3
	Indo-Malayan	98	20
	Nearctic	321	14
	Neotropical	315	76
	Oceania	30	12
	Palearctic	475	14
Short-term (Hansen)	Afrotropical	172	12
	Australasia	42	3
	Indo-Malayan	52	10
	Nearctic	96	2
	Neotropical	127	43
	Oceania	0	0
	Palearctic	196	4

S5. Number of forest populations (generalists and specialists) and forest specialist populations per taxonomic class included in analyses using long-term and short-term tree cover datasets.

Analysis	Taxa	All forest populations	Forest specialist populations
Long-term (Song)	Amphibians	154	19
	Birds	701	73
	Mammals	767	78
	Reptiles	46	5
Short-term (Hansen)	Amphibians	52	2
	Birds	251	33
	Mammals	348	34
	Reptiles	34	5

S6. Number of species trends in the FSI annually, with proportions of positive and negative annual trends

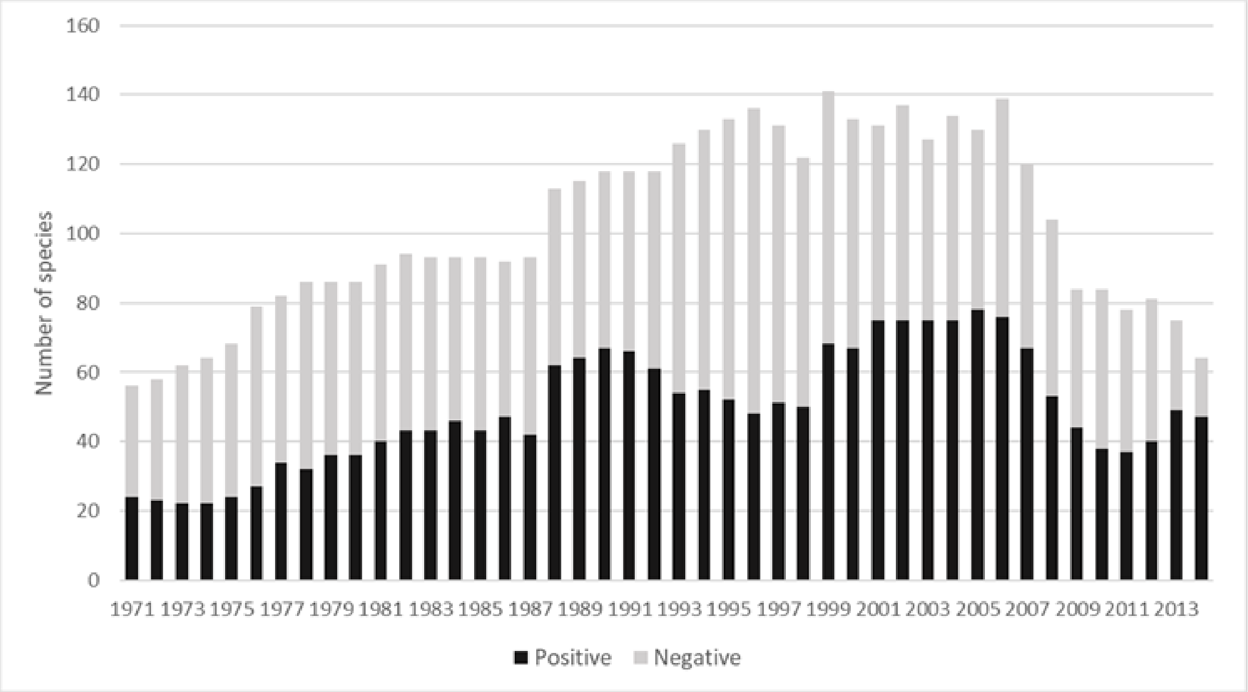

S7. Percentage of annual species trends which are positive or negative for birds (A), mammals (B) and herptiles (C)

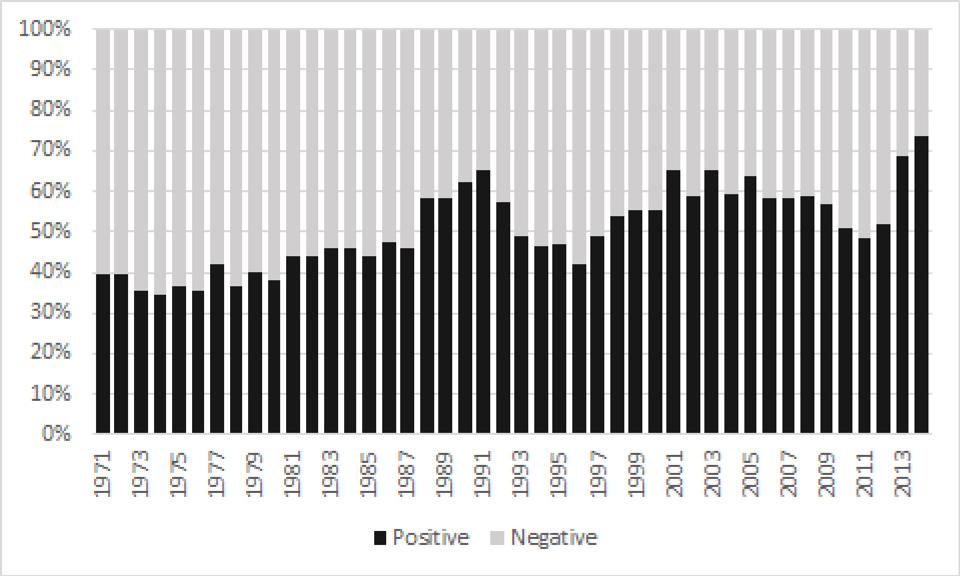

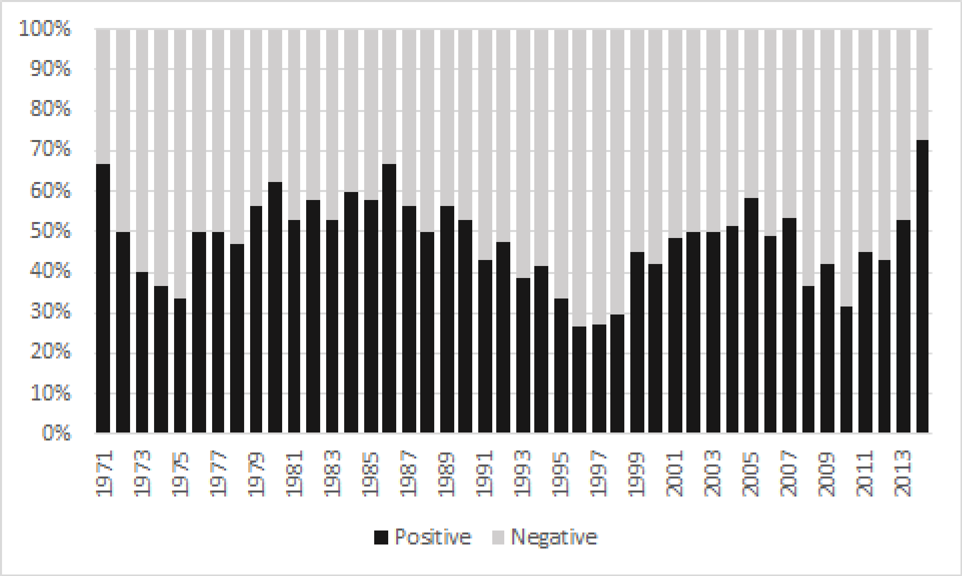

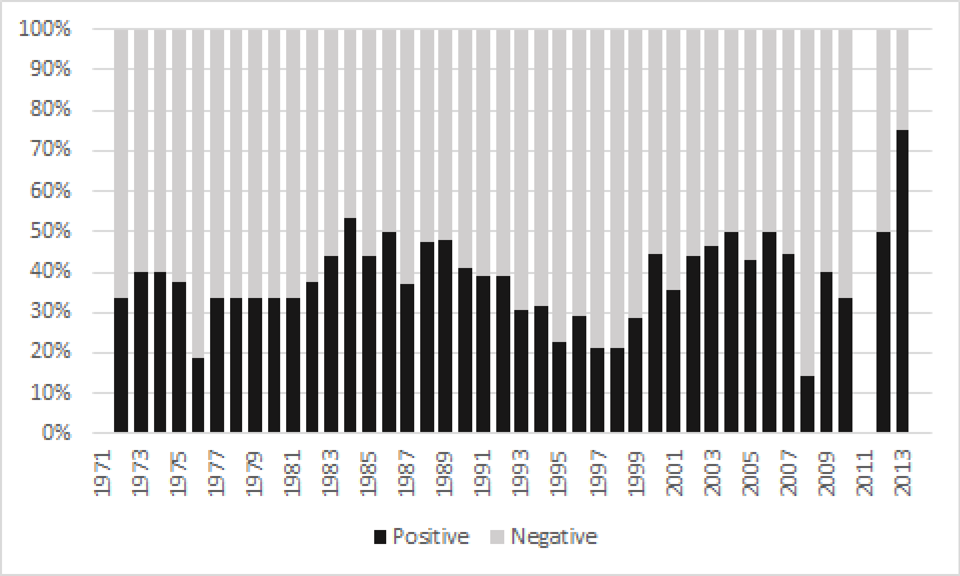
100%
90%
80%
70%
60%
50%
40%
30%
20%
10%
0%
1971
1973
1975
1977
1979
1981
1983
1985
1987
1989
1991
1993
1995
1997
1999
2001
2003
2005
2007
2009
2011
2013
Positive
Negative

S8. Number of average declining, increasing and stable (less than 5% change in abundance) species trends in tropical and temperate realms (A) and biomes (B). Nine species were not located in a forest biome (where forest is the dominant habitat type), so were not included in B.

**A Realm**		**Decline**	**Increase**	**Stable**
**Temperate realms**	Nearctic	22	28	4
	Palearctic	3	7	3
	Total	***25***	***35***	***7***
**Tropical realms**	Afrotropical	18	13	2
	Neotropical	53	48	13
	Indo-Pacific	28	22	4
	Total	***99***	***83***	***19***
**B Biome**		**Decline**	**Increase**	**Stable**
**Temperate forest biomes**	Boreal forests / taiga	2	10	
	Mediterranean forests, woodlands and scrub	1	1	
	Temperate broadleaf and mixed forests	21	24	7
	Temperate coniferous forests	5	4	
	Total	***29***	***39***	***7***
**Tropical forest biomes**	Mangroves	1	2	
	Tropical and subtropical coniferous forests	2	1	
	Tropical and subtropical dry broadleaf forests	9	1	5

	Tropical and subtropical moist broadleaf forests	77	72	14
	Total	***89***	***76***	***19***

S9. Average rate of forest population change regressed on fixed predictor effects with a random effect of location: mixed effects model results for forest vertebrate populations (generalists and specialists). Populations had at least 2 years of data covering at least a 5 year period from 1982-2016. Tree cover variables calculated using the Song et al (2018) dataset. N = 1668.

**Fixed effect**	**Estimate**	**CI**	**AIC**	**ΔAIC to null model**
NULL	*NA*	*NA*	-4113.666	0
Tree cover trend	1.09×10^{-2}	8.43×10^{-4} – 2.09×10^{-2}	-4107.464	+6.202
Body mass	3.92×10^{-3}	1.44×10^{-3} – 6.40×10^{-3}	-4109.732	+3.934
Mean tree cover	-1.91×10^{-3}	-7.45×10^{-3} – 3.65×10^{-3}	-4102.222	+11.444
Mean bare ground	8.79×10^{-4}	-8.66×10^{-3} – 1.04×10^{-2}	-4102.885	+10.781
Exploitation (Y)	1.87×10^{-3}	-1.26×10^{-2} – 1.64×10^{-2}	-4103.753	+9.913

HPD	3.69×10^{-3}	-1.25×10^{-3} – 8.63×10^{-3}	-4103.674	+9.992
Road density	-2.30×10^{-5}	-3.85×10^{-4} – 3.39×10^{-4}	-4096.325	+17.341
Mean travel time	-1.22×10^{-3}	-7.58×10^{-3} – 5.14×10^{-3}	-4102.185	+11.481

S10. Average rate of forest population change regressed on fixed predictor effects with a random effect of location: mixed effects model results for forest vertebrate populations (specialists only). Populations had at least 2 years of data covering at least a 5 year period from 1982-2016. Tree cover variables calculated using the Song et al. (2018) dataset. N = 175.

**Fixed effect**	**Estimate**	**CI**	**AIC**	**ΔAIC to null model**
NULL	*NA*	*NA*	-461.632	0
Tree cover trend	1.86×10^{-2}	-5.19×10^{-3} – 4.25×10^{-2}	-454.989	+6.643
Body mass	-4.03×10^{-3}	-1.17×10^{-2} – 3.69×10^{-3}	-451.438	+10.194
Mean tree cover	-1.82×10^{-2}	-6.40×10^{-2} – 2.76×10^{-2}	-454.560	+7.072
Mean bare ground	2.37×10^{-2}	-1.99×10^{-2} – 6.72×10^{-2}	-454.991	+6.641

Exploitation (Y)	-8.79×10^{-2}	-1.38×10^{-1} – -3.74×10^{-2}	-465.041	-3.409
HPD	-6.68×10^{-3}	-2.48×10^{-2} – 1.15×10^{-2}	-452.625	+9.007
Road density	-1.08×10^{-3}	-3.18×10^{-3} – 1.00×10^{-3}	-448.816	+12.816
Mean travel time	1.34×10^{-2}	-9.01×10^{-3} – 3.58×10^{-2}	-453.894	+7.738

S11. Model results from mixed effects models of average rate of population change of forest generalists and specialists regressed on fixed predictor effects with a random effect of location. Populations had at least 2 years of data covering at least a 5 year period from 2000-2015. Tree cover variables calculated using the Hansen dataset. N = 685.

**Fixed effect**	**Estimate**	**CI**	**AIC**
NULL	*NA*	*NA*	-1479.421
Tree cover 2000	-1.50×10^{-3}	-1.15×10^{-2} – 8.54×10^{-3}	-1468.794
Total tree loss	8.60×10^{-4}	-4.13×10^{-3} – 5.85×10^{-3}	-1467.425
Tree cover change 2000-2010	-2.77×10^{-3}	-2.70×10^{-2} – 2.15×10^{-2}	-1470.524
Body mass	4.82×10^{-3}	7.34×10^{-4} – 8.90×10^{-3}	-1472.199
Exploitation (Y)	1.59×10^{-2}	-1.09×10^{-2} – 4.27×10^{-2}	-1472.025
HPD	9.34×10^{-3}	1.31×10^{-3} – 1.75×10^{-2}	-1473.445
Road density	1.87×10^{-4}	-5.26×10^{-4} – 9.03×10^{-4}	-1463.679
Mean travel time	-6.97×10^{-3}	-1.85×10^{-2} – 4.49×10^{-3}	-1470.392

S12. Model results from mixed effects models of average rate of population change of forest specialists regressed on fixed predictor effects with a random effect of location. Populations had at least 2 years of data covering at least a 5 year period from 2000-2015. Tree cover variables calculated using the Hansen dataset. N = 74.

**Fixed effect**	**Estimate**	**CI**	**AIC**
NULL	*NA*	*NA*	-158.048
Tree cover 2000	-1.90×10^{-2}	-6.88×10^{-2} – 2.97×10^{-2}	-151.075
Total tree loss	2.58×10^{-3}	-1.36×10^{-2} – 1.75×10^{-2}	-148.242
Tree cover change 2000-2010	6.14×10^{-2}	-2.73×10^{-1} – 3.96×10^{-1}	-154.474
Body mass	-1.27×10^{-3}	-1.30×10^{-2} – 1.07×10^{-2}	-147.690
Exploitation (Y)	-5.43×10^{-2}	-1.17×10^{-2} – 8.55×10^{-3}	-153.850
HPD	-2.14×10^{-4}	-1.75×10^{-2} – 1.98×10^{-2}	-148.417
Road density	-1.07×10^{-3}	-3.43×10^{-3} – 1.29×10^{-3}	-145.234
Mean travel time	1.37×10^{-3}	-2.90×10^{-2} – 2.97×10^{-2}	-149.413

S13. Abundance index for 74 species from the FSI with data present throughout all decades from 1970 to 2014 (these are primarily birds from the Nearctic)

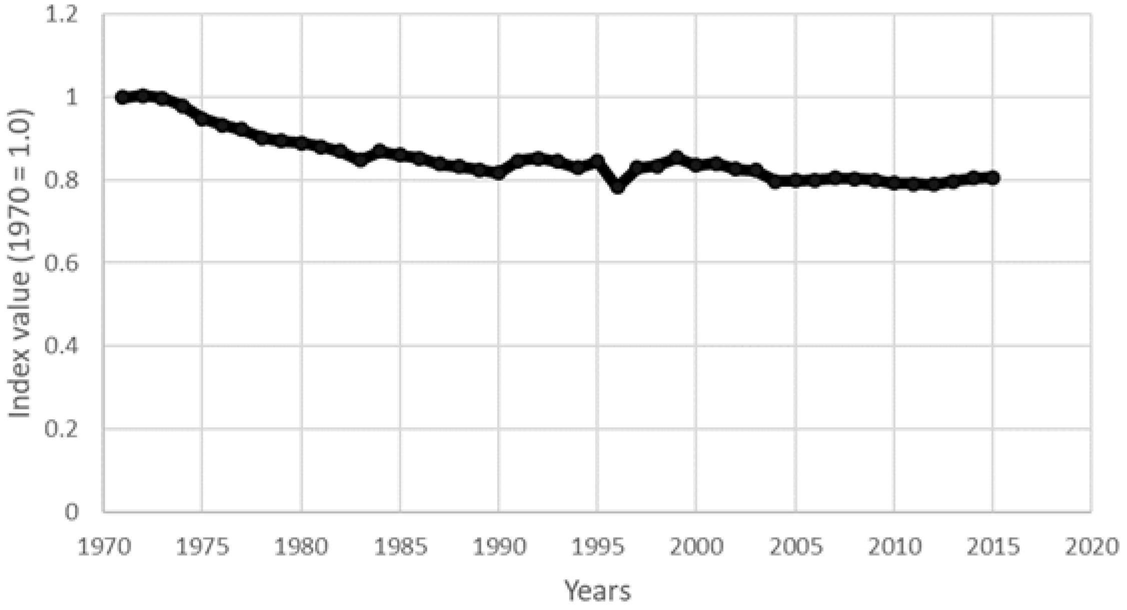

REFERENCES (excluding those cited in the main text)

DiMiceli, C., Carroll, M., Sohlberg, R., Kim, D. H., Kelly, M., & Townshend, J. R. G. (2015). *MOD44B MODIS/Terra Vegetation Continuous Fields Yearly L3 Global 250m SIN Grid V006 [Data set]*. Retrieved from https://doi.org/10.5067/MODIS/MOD44B.006

Sexton, J. O., Song, X.-P., Feng, M., Noojipady, P., Anand, A., Huang, C., … Townshend, J. R. (2013). Global, 30-m resolution continuous fields of tree cover: Landsat-based rescaling of MODIS vegetation continuous fields with lidar-based estimates of error. *International Journal of Digital Earth*, *6*(5), 427–448. https://doi.org/10.1080/17538947.2013.786146
